# Vulcanite Litigation

**Published:** 1874-01

**Authors:** 


### ARTICLE IV.

### *Vulcanite Litigation.*

It will be remembered that it was stated in the November number of the *Dental Cosmos* that a stipulation had been signed between the counsel to complete and try the Massachusetts suit as a test case. We give below a copy of this stipulation.

Under this agreement, the Company having put in their *prima facie* case, the defendant proceeded to put in his proofs and to take testimony in New York and Philadelphia, closing December 17th. The complainants also closed on the 17th of December. The cause has been set for hearing on the 14th of January, before Hon. George F. Shepley, Circuit Judge for the First Circuit.

### UNITED STATES CIRCUIT COURT.

DISTRICT OF MASSACHUSETTS.—Goodyear Dental Vulcanite Company and Josiah Bacon, *vs.* Daniel H. Smith.

DISTRICT OF NEW JERSEY.—Same, *vs.* Charles S. Stockton, same *vs.* Frank A. Cummings.

DISTRICT OF DELAWARE.—Same, *vs* Zenas A. Vandeventer.

EASTERN DISTRICT OF PENNSYLVANIA.—Same *vs.* Robert E. Difenderfer. New Suit, Bill filed. Sept. 1873. Same *vs.* William H. Gates, New Suit, Bill filed. Sept. 1873. Same, *vs.* Wm. Harvey Roop. New Suit, Bill filed, Sept. 1873. Same *vs.* George A. Sinclair. New Suit, Bill filed, Sept. 1873.

---

It is stipulated and agreed that all proofs taken for final hearing in the above entitled suit of Goodyear Dental Vulcanite Company, et al, *vs.* Daniel H. Smith, shall be taken as the proofs for final hearing in each of the above entitled causes, a printed copy of the proofs in said suit taken on behalf of each party, including documentary exhibits, shall be filed with the clerk of the court in each of the above districts within ten days after the proofs are closed on behalf of such party, as the stipulated testimony in the above entitled cause pending in said district, and such testimony shall be read and used at the hearing of each of said causes the same and with the same effect as if the same had been regularly and duly taken in each of said causes separately. Within the same period of ten days, 10 copies ot defendant's proofs shall be delivered to Lee & Alvord, of New York, for the use of the complainants, and within the same time, 10 copies of complainant's proofs shall be delivered to Henry Baldwin, Jr., Esq., of Philadelphia, for the use of the defendant. Said suit *vs,* Smith shall be placed on the calendar for the October Term, 1873, and shall be brought to final hearing before the Circuit Judge of the First Circuit at the earliest day that he will appoint, not less than thirty days after the printed proofs on both sides are interchanged as above provided, and at any place within said Circuit that said Judge may designate; the party proposing to apply for such appointment to give reasonable previous notice to the opposite party of the time and place which are proposed in the application to be made for such appointment. None of the causes above entitled shall be brought to hearing on pleadings and proofs, or otherwise, until after the said Smith suit shall have been decided.

All proceedings in said causes shall be had under due notices, to be served only upon and by Lee & Alvord for complainants, and upon and by Henry Baldwin, Jr., for defendants. Said testimony may be taken before Aubrey H. Smith, Esq., of Philadelphia, Kenneth G. White, Esq., of New York, or John G. Stetson, Esq., of Boston, who are hereby agreed upon as Special Examiners for that purpose. It is understood that the printed copies of the proofs already taken by the complainants may be filed and served within ten days after the signing of this stipulation, dated September 30th, 1873.

[Signed]

LEE & ALVORD,
*Sol'rs for Compl'ts in suits vs. Smith, Stockton and Cummings.*

J. E. SHAW,
*Sol'r for Compl'ts in suits vs. Difenderfer, Gates, Roop, and Sinclair.*

SAMUEL M. HARRINGTON
*Sol'r for Compl'ts in suit vs. Vandeventer.*

---

During the taking of complainants' testimony they issued the annexed subpœna, making the undersigned a witness in the case.

THE PRESIDENT OF THE UNITED STATES OF AMERICA, TO *Samuel S White, Greeting*:

*L. S.*
*U. S. Supb.*

We command you, That, all and singular business and excuses being laid aside, you and each of you be and appear in your persons, before John A. Shields, Esq., special examiner in suit hereinafter mentioned, and a Commissioner appointed by the Circuit Court of the United States of America, for the Southern District of New York, in the Second Circuit, at the office of Lee & Alvord, No. 20 Nassau Street, in the city of New York, in the said Southern District of New York, on the 21st day of November, one thousand eight hundred and seventy three, at 4.20 o'clock in the afternoon of the same day, to testify all and singular what you and each of you may know in a certain cause now depending undetermined in the Circuit Court of the United States for the District of Massachusetts, wherein the Goodyear Dental Vulcanite Company and Josiah Bacon are complainants, and Daniel H. Smith is defendant, on the part of complainants.

And this you or either of you are not to omit, under the penalty upon each and every of you of two hundred and fifty dollars.

Witness, Hon. Nathan Clifford, Associate Justice of the Supreme Court of the United States, at the City of New York, the twenty-first day of November, in the year of our Lord one thousand eight hundred and seventy-three.

[Signed] KENNETH G. WHITE,
*Clerk.*

---

As an element of the history of the attempts to embarrass those who regard the Cummings patent invalid, an action has been commenced against the publisher of this Journal, by a writ served upon him Dec. 10th, 1873, of which the following is a copy.

THE PRESIDENT OF THE UNITED STATES OF AMERICA, TO *The Marshall of the Southern District of New York, Greeting:*

We command you, as we have before commanded you, that you take Samuel S. White, of Philadelphia, Pennsylvania, and a citizen of the State of Pennsylvania, defendant, if he shall be found in your district, and him safely keep so that you may have his body before the Judges of the Circuit Court of the United States of America for the Southern District of New York in the second circuit, to be held at the United States Court Buildings, No. 41 Chambers Street, in the City of New York, in the said Southern District, on the first Monday of January, 1874, to answer unto The Goodyear Dental Vulcanite Company, a corporation created by the laws of New York, and a citizen of the State of New York, plaintiff, in a plea of trespass; and also, to a certain bill of the said plaintiff against the said defendant for a plea of trespass on the case to the damage of said plaintiff in the sum of one hundred thousand dollars, according to the custom of the said court, before the said

Judges, then and there to be exhibited, and that you have then and there this writ.

Witness the Honorable Nathan Clifford, Associate Justice of the Supreme Court of the United States, at the city of New York, the third day of December, in the year one thousand eight hundred and seventy-three, and of the Independence of the United States the ninety-eighth.

LEE & ALVORD,
*Attorneys.*

KENNETH G. WHITE,
*Clerk.*

---

We do not know what trespasses we have committed upon any rights of the Company bringing this action, but will doubtless be informed by the specification of their charges when filed.

The further progress of these cases will be duly reported in the *Dental Cosmos.*

SAMUEL S. WHITE.

